# Behavioral, Endocrine, and Neuronal Responses to Odors in Lampreys

**DOI:** 10.3390/ani15142012

**Published:** 2025-07-08

**Authors:** Philippe-Antoine Beauséjour, Barbara S. Zielinski, Réjean Dubuc

**Affiliations:** 1 Department of Neurosciences, Faculty of Medicine, University of Montreal, Montreal, QC H3T 1J4, Canada; philippe-antoine.beausejour@umontreal.ca; 2 Department of Integrative Biology, Faculty of Science, University of Windsor, Windsor, ON N9B 3P4, Canada; zielin1@uwindsor.ca; 3 Research Group in Adapted Physical Activity, Department of Exercise Sciences, Faculty of Sciences, University of Quebec in Montreal, Montreal, QC H2X 1Y4, Canada

**Keywords:** lamprey, olfaction, chemosensory signaling, pheromones, behavior, olfactory ecology, endocrinology, reproduction, neuroscience, sensorimotor integration

## Abstract

Lampreys are primitive fish that rely significantly on olfactory cues throughout their complex life cycle. The olfactory system of the sea lamprey (*Petromyzon marinus*) is among the best characterized in vertebrates. In recent decades, tremendous advances have been made by isolating individual compounds from sea lampreys that can replicate natural behavior when artificially applied in the wild. In no other aquatic vertebrate has the olfactory ecology been described in such extensive detail. In the first section, we provide a comprehensive review of olfactory behaviors induced by specific, individual odorants during every major developmental stage of the sea lamprey in behavioral contexts such as feeding, predator avoidance, and reproduction. Moreover, pheromonal inputs have been shown to induce neuroendocrine responses through the hypothalamic-pituitary-gonadal axis, triggering remarkable developmental and physiological effects, such as gametogenesis and increased pheromone release. In the second section of this review, we describe a hypothetical endocrine signaling pathway through which reproductive fitness is increased following pheromone detection. In the final section of this review, we focus on the neuronal circuits that transform olfactory inputs into motor output. We describe specific brain signaling pathways that underlie odor-evoked locomotion. Furthermore, we consider possible modulatory inputs to these pathways that may induce plasticity in olfactory behavior following changes in the external or internal environment. As a whole, this review synthesizes previous and recent progress in understanding the behavioral, endocrine, and neuronal responses of lampreys to chemosensory signals.

## Simple Summary

Lampreys are primitive, jawless fish that rely extensively on their sense of smell to guide behavior. This review explores how chemical signals influence their development, reproduction, and survival. The first section reviews how lampreys use odors at different life stages to locate food, avoid predators, migrate to habitats suitable for reproduction, and select a mating partner. The second section focuses on how scent detection triggers developmental changes such as sexual maturation. We describe a hypothetical signaling pathway through which specific chemicals, known as pheromones, activate the neuroendocrine system, which in turn influences the levels of sex hormones, leading to the physical and behavioral changes necessary for reproductive fitness. The final section delves into neuronal mechanisms, exploring how olfactory stimuli trigger behavioral responses and describing specific neuronal pathways that process odor signals from the nasal cavity to the brain and then to spinal cord neurons that control locomotion. Studying lampreys offers a unique insight into the evolution of sensory systems and brain function. By synthesizing previous and recent findings, this review enhances our understanding of olfaction in lampreys. It provides a valuable and comprehensive summary suitable for researchers in the fields of biology, ecology, ethology, endocrinology, and neuroscience.

## 1. Introduction

Lampreys hold a pivotal phylogenetic position as they belong to the most ancient extant lineage of vertebrates, known as the Agnatha. The sea lamprey, *Petromyzon marinus*, is a parasitic fish that invaded the Great Lakes of North America and preys on large-sized fish. Its presence has caused significant damage to the fishing industry, leading to extensive research into lamprey behavior, which was found to be strongly biased by odorants. As a result, the olfactory ecology of the sea lamprey has been studied in-depth for decades and is now characterized to an unparalleled extent compared to any other aquatic vertebrate. Indeed, many individual chemicals have been isolated from lampreys and can induce stereotypical behavior when artificially applied in the environment. This review provides a comprehensive description of the following topics: (1) the behavior of lampreys at different life stages in response to various odorants found in their natural habitat; (2) endocrine signaling mechanisms activated by the detection of pheromonal compounds that induce developmental changes by acting on the hypothalamic-pituitary-gonadal (HPG) axis; (3) neuronal signaling mechanisms within the central nervous system that elicit behavior following exposure to salient odorants.

## 2. Olfactory-Induced Behavior in Lampreys

**Figure 1:**
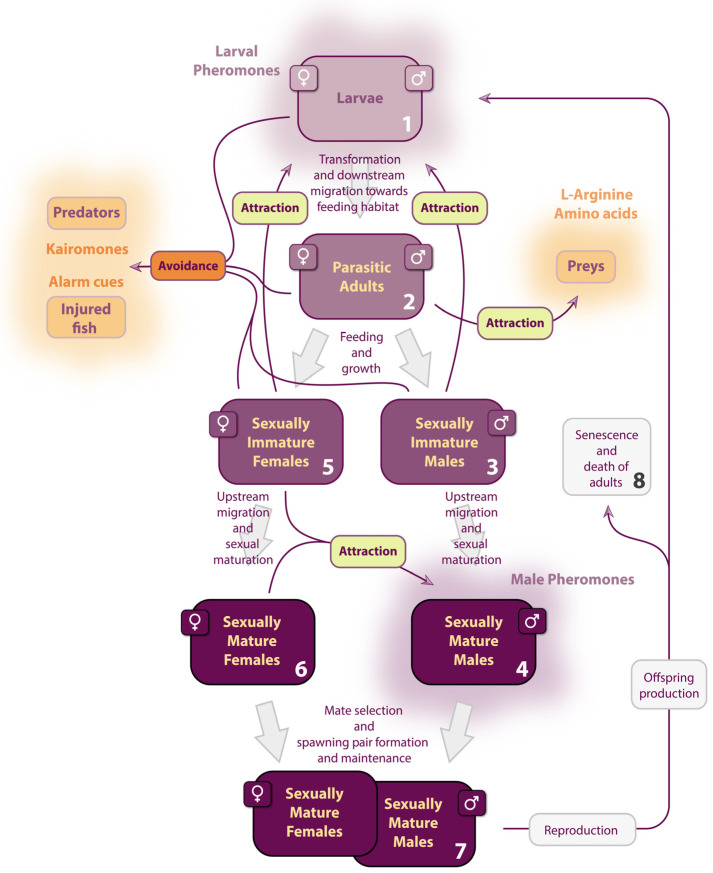
A pictorial ethogram of the sea lamprey’s olfactory behavior. The schematic illustration depicts the various developmental stages of the sea lamprey and the different odorants that induce olfactory behaviors throughout its life cycle. After birth, (1) larvae are mostly sedentary but avoid predators when detecting anti-predator cues such as kairomones from predators and alarm cues from injured fish, including conspecific and heterospecific lampreys, and other sympatric non-lamprey fish. These anti-predator cues also drive predator avoidance in parasitic adults and sexually immature, upstream-migrating lampreys of both sexes, but not in sexually mature adults. Following metamorphosis, (2) parasitic adults feed on prey fish whose body odor contains attractive amino acids. After 12 to 18 months of parasitic feeding, (3) sexually immature males migrate upstream to spawning sites, guided by larval pheromones. After completing their migration, the now (4) sexually mature males emit sex pheromones that contribute to guiding (5) upstream-migrating sexually immature females to spawning sites, in addition to the larval migratory pheromones. After completing their migration, the now (6) sexually mature females are no longer attracted to larval pheromones. They are strongly biased toward male sex pheromones, which are essential for (7) spawning pair formation and maintenance. Shortly after breeding, (8) spent males and females die of senescence and their offspring develop into (1) the larval stage, thus completing the life cycle.

The sea lamprey’s life cycle is distinctive, encompassing larval, parasitic juvenile, and reproductive adult stages. Initially, sea lampreys filter-feed as larvae for several years before undergoing metamorphosis into parasitic juveniles. At this point, they prey on fish to augment their body mass. Subsequently, adults migrate to spawning grounds to reproduce, culminating in their death. Lampreys are predominantly nocturnal animals inhabiting aquatic environments characterized by low visibility, and they exhibit solitary behavior, lacking social structures such as shoaling [1,2,3]. Throughout their life cycle, they thus rely significantly on chemosensory cues to make critical choices related to survival and reproduction (Figure 1). Accordingly, lampreys possess a highly developed olfactory apparatus capable of detecting molecules in the picomolar range, enabling them to track odor plumes in their underwater habitats. Consequently, lampreys can chemically detect fish such as preys and predators, and of course, other lampreys. Among the 41 species of lampreys, many co-exist in the same geographic areas, employing shared signaling mechanisms due to overlapping predator dynamics and habitat preferences for rearing and spawning. This review aims to synthesize the existing knowledge regarding olfactory-induced behavior throughout the life cycle of the sea lamprey, *Petromyzon marinus*.

### 2.1. Larval Stage

Sea lamprey eggs hatch in nests built within the gravel beds of riverine streams, where pro-larvae develop for a period of 17 to 33 days, ultimately reaching Piavis stage 17 [4]. At this stage, the lamprey can swim and leaves the nest even before its eyes fully develop. However, the pro-larval lamprey is not entirely blind to its surroundings; its olfactory mucosa contains chemosensory neurons that respond to various amino acids, bile products, and water conditioned by conspecifics [5]. Consequently, the olfactory organ likely plays a crucial role early in life, as pro-larval sea lampreys possess well-developed olfactory systems [5,6]. After emerging, sea lampreys migrate to downstream silted areas, burrow into fine sediments and subsist on organic detritus for many years as filter-feeding larvae [7,8]. The attraction of larvae to rotting potato haulms placed in the stream provides evidence that chemosensory cues are used to locate food during this stage [7,9]. Moreover, larvae are particularly vulnerable to predation, not only by fish [10], but also by amphibians, reptiles, birds, and mammals [11]. Burrowing into sediments protects from predators [12], leading larvae to adopt a largely sedentary lifestyle while gradually dispersing downstream [13,14]. To reduce predation risks, larvae rely on the chemosensory detection of soluble substances in their aquatic environment. They respond to chemical cues emitted by injured lampreys, injured heterospecific fish, or predators, all of which are reliable indicators of predation risk, triggering anti-predator behaviors. In laboratory experiments [15], exposure to extracts from conspecific lampreys or heterospecific fish increased both the rate of escape attempts and direction changes, often exhibited as “zig-zag” movements, similar to anti-predator behaviors observed in other prey fish [16]. Furthermore, if sand is present at the bottom of the experimental environment, exposure to conspecific lamprey extract or predator extract significantly reduces downstream drift [14,17]. This suggests that anti-predator cues may signal a predatory event, prompting larvae to remain within their protective burrows.

### 2.2. Metamorphosis and Juvenile Stage

Larvae undergo a metamorphosis that results in significant changes in body structure, allowing juvenile sea lampreys to thrive as hematophagous ectoparasites feeding on larger fish. The morphological differences are so pronounced that larvae were initially described as a separate species, *Ammocœte branchialis* [18], and continue to be referred to as “ammocœtes” today. Following metamorphosis, critical alterations are observed in the peripheral olfactory system [6]. Notably, extensive neurogenesis and differentiation of olfactory sensory neurons [19] lead to dramatic modifications in the shape and mass of the nasal sac, which doubles in relative weight [20]. This growth, in conjunction with the development of eyes and a sucker mouth, has significant behavioral implications, enabling newly transformed juvenile sea lampreys to immediately begin parasitic feeding in their natal stream [21,22,23].

At the onset of their juvenile stage, sea lampreys migrate downstream to feed in lake or sea habitats. Like larvae, these migratory juveniles escape potential predators by avoiding chemicals released from injured lampreys, but do so by accelerating their downstream movement [24]. How do sea lampreys effectively locate and attach to larger fish? Within a 200 mm radius, they generate a pulsating electrical field around their head region that may facilitate prey detection by electrolocation [25,26]. However, long-distance orientation toward prey is predominantly reliant on olfactory cues. Indeed, locomotor activity in juvenile sea lampreys significantly increases upon exposure to water containing the composite odor of trout [27]. This stimulus not only enhances locomotion but also attracts lampreys, which strongly prefer areas perfused with trout-conditioned water in compartmentalized experimental tanks. Blocking the nasal tube abolishes these behaviors, suggesting a clear dependence on olfaction. Interestingly, these results were reproduced by using a single amine isolated from trout-conditioned water: arginine.

Among the various amino acids detected by sea lampreys, L-arginine stands out as the most potent, detectable at exceptionally low concentrations (10^−10^ M) [28,29]. As an essential amino acid in fish, arginine is crucial for numerous metabolic processes [30]. Many fish species, including important prey for sea lampreys such as salmon [31] and trout [32,33], have particularly high requirements for arginine compared to other essential amino acids [34]. Moreover, L-arginine is also the direct precursor of nitric oxide, a very potent transcellular and intracellular signaling molecule, and the sea lamprey’s responsiveness to L-arginine was shown to depend at least partially on its enzymatic reduction by nitric oxide synthase in the main olfactory epithelium (MOE) [29]. Since the MOE of sea lampreys is a million times more sensitive to L-arginine than to other amino acids [28], this molecule likely plays a pivotal role in guiding long-distance predatory behavior. Other amino acids detected at higher concentrations may serve for close-range localization, identification of prey, or other functions yet to be elucidated.

### 2.3. Male Upstream Migration

The growth rate during the larval, filter-feeding stage is relatively low, requiring several years to reach just a few grams. In contrast, adult sea lampreys can exceed two kilograms after 12 to 18 months of parasitic feeding as juveniles [35]. During this period, atrophy of the gut and progressive gonadal development lead to the cessation of feeding and the onset of the adult stage, as well as spawning migration [36]. Lampreys face numerous challenges in locating suitable habitats for their final act: reproduction. Since they rely on a single mating opportunity in their lifetime, the ability to congregate in appropriate spawning grounds is crucial. Although lampreys are solitary animals, they have evolved a complex communication system that facilitates synchronized gathering for reproduction. Timing is particularly vital, as breeding events are seasonal and adult sea lampreys must rely on stored nutrients accumulated during their parasitic juvenile stage until their death, which occurs shortly after spawning [37]. In land-locked sea lampreys of North America, the locations for gathering are also limited: among the tributaries of the Great Lakes, which contain one-fifth of Earth’s surface freshwater, only 7.5% have supported larval growth [10]. The scarcity of such habitats in the Great Lakes highlights the need to identify suitable spawning habitats accurately.

In this migratory journey, chemosensory signals play a pivotal role. Unlike other migratory fish, such as salmonids that learn the chemical composition of their birthplace, sea lampreys do not return to their natal streams [38,39,40]. Instead, they exhibit regional panmixia by employing a “suitable river” strategy, involving olfactory assessment of spawning habitats based on contemporaneous chemical signaling [40,41]. Initially, males must navigate open water to locate an appropriate stream. They conduct an extensive search along the shoreline until they encounter river water [42], which they prefer over lake water [43]. Upon detection of river water, they shift to an intensive stream-finding search and orient toward the river mouth, guided by chemical signals present in the river plume [44,45,46]. The unidirectional flow of water, constrained within the channel, allows upstream odor sources to “activate” the entire river discharge and bias stream selection during migration [47,48,49]. Notably, when sea lampreys are experimentally rendered anosmic, they fail to locate rivers [50]. In contrast, artificial blinding does not affect their migratory behavior [2].

The olfactory cues that indicate the presence of an upstream habitat suitable for spawning encompass two types of signals: (1) attractive migratory pheromones and (2) repulsive anti-predator cues.

#### 2.3.1. Attractive Larval Migratory Pheromones

Bile products specific to the sea lamprey [51] are excreted by stream-resident larvae [52] and attract adults, prompting them to migrate upstream [53,54]. These bile products are synthesized in the liver, stored in the gall bladder, and subsequently excreted via the intestine along with feces. As a by-product of larval feeding, migratory pheromones are released at higher rates by well-fed larvae [55] and signal the presence of high-quality reproductive habitats [44,47,56,57] that are conducive to spawning and the successful rearing of offspring. These odorants diffuse effectively through large volumes of turbid water, released at rates sufficient to produce biologically relevant concentrations in river water (in the picomolar range) [58] and degrading slowly enough for the entire pheromone to persist at river mouths [55]. At nanomolar concentrations, larval bile products elicit strong electrophysiological responses in MOE [59], enhance swimming activity, and guide adult males upstream [60]. Hence, the smell of well-fed larvae attracts upstream-migrating adults into rivers suitable for reproduction.

An initial characterization of the individual compounds comprising the larval migratory pheromone led to the identification of its primary component: 3-keto petromyzonol sulfate (3kPZS), a bile product specific to lampreys that is detected by downstream migratory adults and induces long-distance upstream navigation to habitats capable of supporting larval rearing [44,61,62,63]. Moreover, larvae release two additional compounds, petromyzonol sulfate (PZS) and allocholic acid [55,58,59,64,65], that induce low threshold responses in the MOE [55,59,64,66], increase swimming activity [60], and elicit weak attraction [43] in migratory sea lampreys. While PZS plays a role in the behavioral response to larval odor, demonstrating distinct effects in migratory versus sexually mature adults (see Section 2.4.2), allocholic acid has no observable behavioral effects [65,67].

Further analysis of pheromonal compounds in larval odor led to the identification of two other bile products—petromyzonamine disulfate and petromyzosterol disulfate—both of which stimulate the MOE and attract migratory sea lampreys in laboratory maze assays [55,67,68,69,70]. Moreover, field assays at river mouths demonstrated that petromyzonamine disulfate and petromyzosterol disulfate raise the time migratory sea lampreys spend in river plumes, potentially facilitating their search for river entrances [45]. However, these compounds do not increase entry rates into the river [45] or upstream movement within the river [71]. Interestingly, when PZS is combined with petromyzonamine disulfate and petromyzosterol disulfate, the resulting mixture is as attractive as the entire larval odor in laboratory settings [67], and enhances the upstream movement induced by 3kPZS in migratory sea lampreys [63]. Despite this, the mixture fails to trigger the same behavioral effects as the complete larval odor in natural habitats [45,63,71], prompting ongoing investigations to uncover previously unidentified molecules that may act as larval migratory pheromones. Noteworthy candidates include petromyzonin (a sulphated hexahydrophenanthrene) [72], petromyroxols (fatty acids) [73,74], and petromyric acid A (a fatty acid) [75], all of which stimulate the MOE of adult sea lamprey and could putatively induce behavioral bias in stream selection. Among these larval products, behavioral assays were performed only on petromyric acid A, which guides migratory females in the selection of tributaries within a river [75]. These studies indicate that the larval migratory pheromone contains multiple compounds that guide distinct behaviors; however, the full extent of these bioactive molecules has not been entirely identified to date.

Land-locked sea lampreys share habitat preferences with various lamprey species [76] that spatially overlap in their geographic distribution [77]. Interestingly, heterospecific lampreys use common signaling molecules to facilitate their upstream migration [78]. Indeed, adult sea lampreys respond to migratory pheromones emitted by the larvae of other species [64], which enables them to assess more accurately habitat quality and navigate toward syntopic rearing habitats that can support multiple lamprey populations [11]. In addition to sea lampreys [43,44,54,55,64,66,68], attraction to larval conspecifics occurs in other lamprey species, including silver lamprey [64], river lamprey [79], and Pacific lamprey [80].

#### 2.3.2. Repulsive Anti-Predator Cues

Anti-predator cues represent a second category of chemical signals that guide upstream migration. For the purposes of this review, these cues are classified into two main types: (1) chemicals released from dead or injured animals, known as alarm cues or necromones, and (2) chemicals emitted by potential predators, referred to as kairomones. Released into the aquatic environment, anti-predator cues are publicly available and trigger specific anti-predator behaviors; for an extensive review, see [81]. These signals are crucial for stream selection, enabling lampreys to indirectly assess predation risks before entering a stream [82]. During upstream migration, sea lampreys transition from predator to prey and become especially vulnerable, as they are confined to the river channel and forced to swim through increasingly narrow and shallow waters. As a result, they face constant threats from various predators, including mammals, birds, water snakes, and fish [10,83,84,85,86]. However, the ability to detect anti-predator cues through chemosensory mechanisms allows sea lampreys to reduce predation risks during their spawning migration.

Lamprey alarm cues, which are detected by the MOE [87], elicit anti-predator behaviors such as avoidance and increased activity levels. In experimental tanks, migrating sea lampreys exposed to alarm cues display heightened movement speed, frequent darting, sharp turns, and surface breaches [88,89]. However, when sea lampreys first enter a river from a lake, the presence of high concentrations of alarm cues within the river plume does not prevent their entry [90]. This suggests that at the river mouth, alarm cues not only indicate the likelihood of predation occurring in the river but also signal the presence of larvae and potential mates upstream [90]. Indeed, high concentrations of alarm cues within the river plume promote the entry of migratory sea lampreys, but also accelerate upstream movements, possibly minimizing time spent in risky areas [90].

In rivers where larval migratory pheromones are present, behaviors differ, as upstream migrants reduce their risk exposure by avoiding areas scented with alarm cues when safe spaces devoid of such cues are available. Alternatively, when the entire channel is activated with alarm cues, they increase the upstream movement speed [87,91,92,93,94,95,96]. Moreover, upstream migrants tend to avoid entering tributaries scented with alarm cues [94]. Interestingly, during the daytime, immobile sea lampreys show no response to chemosensory alarm cues [97]. Hence, during migration in rivers activated by larval odors, alarm cues do not affect whether lampreys swim upstream, but they do impact movement timing, speed, and path to minimize predation risk [87].

Given that sea lampreys share common predators with other species of lampreys [98], they exhibit avoidance and flight responses to alarm cues released from injured heterospecific lampreys [85,92,99]. Moreover, strong avoidance responses have also been observed following exposure to alarm cues from other fish species that coexist with sea lamprey populations, such as *Catostomus commersonii* [100].

Kairomones, including human saliva (a mammalian kairomone) [101], washings from the Northern water snake (*N. sipedon*, a reptilian kairomone), and 2-phenylethylamine (a biogenic amine found in the urine of mammalian carnivores) [102], provoke anti-predator behaviors in lampreys [94,95,97,100,103,104]. The intensity of avoidance behaviors in response to repulsive anti-predator cues increases with the concentration of the stimulus [87,92,95]. Moreover, habituation, defined as a decrease in avoidance behaviors, occurs when sea lampreys are continuously exposed to a fixed concentration of alarm cues for extended periods (2 to 4 h) and cannot avoid exposure [104,105]. Conversely, habituation does not manifest when sea lampreys are subjected to intermittent exposure for shorter durations (20 min) [103] or when they can freely navigate in and out of the odor plume [105]. Furthermore, habituation also follows exposure to alarm cues from other fish like *Catostomus commersonii*, as well as to kairomones such as human saliva [103]. Interestingly, applying a mixture of both alarm cues and kairomones results in enhanced avoidance behaviors [95,100,103]. In this context, alarm cues may indicate that a conspecific or closely related animal has recently been injured, while kairomones signal the proximity of a potential threat. The combination of both provides more reliable evidence of active predation on lampreys.

Identifying the chemical constituents of the lamprey alarm cue that trigger anti-predator behavior poses a significant challenge [106], and only a limited number of individual compounds have been characterized to date. In the skin, water-soluble nitrogenous compounds, including amino acids [89,106,107,108], as well as chloroform-soluble compounds, such as lipids [106], have been shown to induce avoidance behavior in upstream migrants. Among the individual compounds isolated from the water-soluble fraction [106,108], only isoleucine, an amino acid, has been demonstrated to elicit avoidance behavior on its own [106]. Notably, isoleucine also induced avoidance when mixed with a combination of creatinine, arginine, and valine, but not when included in a blend with tyrosine and hypoxanthine [106]. The alarm cue may thus consist of multiple active components that must be present in specific ratios to trigger anti-predator responses effectively.

Additionally, the polyamine putrescine, which has been identified in both sea lamprey skin extracts [108] and human saliva [109], was found to induce avoidance behavior in upstream migrants, although less effectively than the alarm cue blend derived from whole-body or skin extracts [89]. Putrescine, commonly found in decomposing animal tissue, is particularly repulsive to many prey species [110]; however, lampreys may perceive it as an odor of death or decay that signals a disease threat rather than a direct predation risk [89]. Importantly, the alarm cue is not exclusive to skin tissue, as it appears to be distributed throughout the body [92]. Indeed, whole-body extracts and skinless carcasses also elicit avoidance behavior, highlighting the broader distribution of these chemical cues [88,89,92,106].

### 2.4. Female Upstream Migration

After many weeks of upstream migration, male sea lampreys become sexually mature as they reach the spawning area, where they build and aggressively defend a nest [111,112]. To attract females over long distances to their nests, males release a multicomponent pheromone [52,113,114]. The main component of this pheromone is 3kPZS, which is interestingly also the main component of the larval migratory pheromone. As sexually mature adults, male sea lampreys excrete 3kPZS again to draw potential mates upstream. However, in adults, 3kPZS is released at significantly higher rates (larvae: 38 ng per larva per hour; sexually mature males: 816,000 ng per adult per hour; [115]) and through a different mechanism. During metamorphosis, the gallbladder and bile ducts degenerate and are absent in adults [116]. Consequently, 3kPZS is now released from the gills [117] via a complex process of biosynthesis and excretion that occurs after sexual maturation [118,119]; see Section 3.2.2. Released first during the larval stage and then after sexual maturation, 3kPZS plays a crucial role in guiding upstream migration in sexually immature adults toward their spawning grounds.

#### 2.4.1. Identifying a Suitable River

Lampreys inhabit syntopic reproductive habitats that sustain multiple species [11], resulting in the co-occurrence of heterospecific populations that also emit odors and thus contribute to the downstream chemical blend. Chemical profiling of odors released by adult males of various species has revealed considerable overlap in the compounds associated with reproduction, including the notable presence of 3kPZS [120]. Electrophysiological and behavioral tests have confirmed the existence of interspecific responses to lamprey pheromones. Remarkably, female sea lampreys can detect and respond to the odors of adult males from every lamprey species tested [120], despite the observed interspecific variations among male scents [121]. Consequently, at the river mouth, sexually immature female lampreys are stimulated by a mixture of heterospecific larval [64] and adult male [120] compounds. This chemical information represents an authentic signal of successful spawning and rearing [44,85], enabling female sea lampreys to make more accurate olfactory assessments regarding habitat quality and the presence of sexually mature males.

As females follow males upstream in search of reproductive partners, they avoid predation by using the aforementioned anti-predator cues. However, these responses attenuate after sexual maturation [92], which occurs during migration. Upon approaching their nesting habitat, sexually mature females encounter a blend of odors from multiple generations of larval lampreys that have drifted downstream from previous reproductive events and may even inhabit areas mixed with spawning grounds [11]. In this context, females face another olfactory challenge. While they are attracted to 3kPZS released by both larvae and potential breeding partners, they must distinguish between the two in order to successfully complete their migration to spawning grounds.

#### 2.4.2. Locating Spawning Grounds

In sexually immature females, larval bile products function as migratory pheromones [60], whereas in sexually mature females, bile products from mature males act as mating pheromones [52]. It has been proposed that following sexual maturation, females exhibit distinct olfactory responses to these pheromones [122,123]. Field studies have shown that sexually immature females are attracted to both larval and male odors; however, they display a preference for the natural larval pheromone over the male counterpart [124]. This preference may be attributed to additional attractive components present in the larval odor [68,75]. In contrast, field and laboratory experiments have demonstrated that sexually mature females show no attraction to the larval odor, instead being strongly attracted to the odors of sexually mature males [60,115].

The mechanism underlying this behavior involves larvae excreting not only 3kPZS, but also high concentrations of PZS that repel sexually mature females. This repulsion likely prevents females from being misoriented toward larval odors, enabling them to locate mates more effectively [115]. The MOE of sea lampreys contains specialized mechanisms for the detection of both PZS and 3kPZS [59,66,125]. Interestingly, PZS has been shown to reduce responses to 3kPZS within the MOE of female sea lampreys through an unknown mechanism [67,115,125,126]. Accordingly, this reduction in response also lessens the behavioral preference of sexually mature females for 3kPZS, both in laboratory settings and in natural habitats [115]. While both male and larval sea lampreys excrete 3kPZS and PZS, they do so in significantly different proportions: the male-typical ratio is 100:1 (3kPZS/PZS), and the larva-typical ratio is 1:10. This disparity in the relative abundance of each component enables females to discriminate robustly between male and larval odors. In fact, Buchinger et al. [115] demonstrated that mixtures of 3kPZS and PZS at typical ratios could replicate the behavioral responses to natural odors, as sexually mature females consistently chose streams and nests treated with male 3kPZS/PZS ratio over those with larval 3kPZS/PZS ratio.

The sensitivity of the MOE in female adults is reported to be one hundred times higher for 3kPZS than for PZS [125], and a 1:1 ratio of 3kPZS to PZS is required to neutralize the response to 3kPZS [115]. Further evidence supporting the importance of the 3kPZS/PZS ratio involves a synthetic analog of 3kPZS, known as petromyzonol tetrasulfate (3sPZS), that is also detected by the MOE, where it diminishes responses to 3kPZS, significantly affecting female attraction to male odors [127]. This compound acts as a behavioral antagonist: 3sPZS diminishes attraction and spawning behavior induced by 3kPZS or the complete male pheromone in sexually mature females. Moreover, a highly concentrated mixture of 3sPZS and PZS dramatically decreases spawning activity in natural sea lamprey populations, suggesting that the balance of olfactory inputs from both 3kPZS and PZS is critical for reproduction [127]. Thus, sexually mature female sea lampreys respond specifically to a ratio of compounds that provide cues for male location and selection.

In contrast, PZS fails to decrease the locomotor response to 3kPZS in sexually immature females [63]. Unlike sexually mature females [115], sexually immature females do not discriminate between the male-typical ratio of 3kPZS to PZS (100:1) and the larva-typical ratio (1:10); they are attracted to both [124]. This characteristic enables sexually immature females to track both larval and male odors during their upstream migration [124] until they reach sexual maturity and are ready to mate.

Interestingly, the sensitivity of the MOE to larval bile products gradually declines as migration progresses [128]. Furthermore, exposure to 3kPZS alone elicits different behavioral responses in females, depending on their stage of sexual maturation. In sexually mature females, 3kPZS induces attraction to the source at close range, prompting entry into baited nests [61,113,114,126,129]. In contrast, sexually immature females exhibit an increase in swimming speed and general search behavior, but do not locate the odor source [93,130]. Thus, sexual maturation significantly alters the behavioral responses of female sea lampreys to pheromones (see Section 4.2.3).

Like females, males also display aversive behavioral responses to PZS after sexual maturation, which may prevent them from orienting toward larvae during nesting and spawning [115]. This response enables males to benefit from this pheromonal cue and avoid tracking larval 3kPZS in fine sediment habitats that are unsuitable for nest building. Consequently, 3kPZS acts as an attractive migratory pheromone (the “Go” signal) that guides immature adults upstream to appropriate spawning grounds. However, because nursery and spawning habitats are intermingled in streams, 3kPZS from larval sources could potentially disrupt reproductive behavior. In this context, PZS serves as a repulsive cue (the “No-Go” signal), keeping sexually mature adults away from nurseries. Together, these lamprey-specific cues function as olfactory traffic lights, efficiently guiding individuals through their riverine journey and facilitating the timely congregation of sexually mature adults in one of the rare tributaries of the Great Lakes that sustain productive spawning sites.

### 2.5. Olfactory Spawning Behavior

During reproductive events, hundreds of sea lampreys synchronously ascend specific rivers to breed [131]. Within the spawning lek, males establish and defend their own nesting territories while females visit multiple nests over the course of approximately one week, after which adults rapidly die of senescence [76]. Actively breeding pairs form and spawn intermittently, roughly every five minutes. Although monogamous pairings have been observed [112], sea lampreys predominantly exhibit polygynandrous behavior [76] and the sex ratios within the spawning population determine the number of females at each nest [132,133]. Notably, the spawning season and territory of the sea lamprey coincide with those of four other lamprey species in the upper Great Lakes [76]. While other lamprey species frequently share nests and can engage in mass spawning [134] without antagonistic behavior between species [135], neighboring male sea lampreys display competitiveness, especially when joined by a female, and viciously evict male intruders from their nesting territories using their sucker mouths [112]. In this disorderly breeding scenario, olfaction becomes crucial for identifying suitable sexual partners.

Olfaction plays a vital role at this stage, as sexually mature females must select appropriate mates amid competing males. Females that are artificially anosmic do not spawn [127], being unattracted to male odors in laboratory conditions and unable to detect sexually mature males in natural stream environments [126]. After being guided upstream by a collective stream odor, these females find themselves at a much closer range, where the scents of individual males become accessible. There is significant variability in the odorant compounds among males [121]. In addition to 3kPZS, males release molecules at various rates that are hypothesized to function as minor components of the mating pheromone and to act as proximity cues—short-range individual pheromones, as opposed to long-range collective migratory pheromone. These putative proximity cues are detected by the MOE of sexually mature females, who demonstrate a preference for specific ratios of these pheromonal blends. However, the complete spectrum of molecules present in male odors remains to be fully elucidated. Short-range pheromones may provide several evolutionary advantages during mate selection and spawning, as they likely function in close-range assessment, enabling females to confirm male identity, proximity, and reproductive readiness before spawning [136,137].

#### 2.5.1. 3-Keto Petromyzonol Sulfate

Among the various putative pheromone compounds identified in the washings of sexually mature male sea lampreys, the majority are bile products. The most prominent among these is 3kPZS, recognized for its role in eliciting migratory behavior and considered as the principal component of the male pheromone. Beyond facilitating long-range migration and the congregation of multiple lamprey species at spawning grounds, 3kPZS is also essential to near-source courtship behaviors. It significantly influences the behavior of sexually mature females by enabling nest localization and fostering the selection of preferred nesting sites [114]. Additionally, it prompts nest construction, nest cleaning, and pair maintenance behaviors [114,129]. However, while 3kPZS induces upstream movements comparable to those prompted by the complete male odor, sexually mature females prefer the latter [49]. Moreover, 3kPZS alone fails to elicit the full range of sexual behaviors and is less effective than the complete male odor at retaining females within nests [113,114,129,138,139,140].

#### 2.5.2. 3,12-Diketo-4,6-petromyzonene-24-sulfate

The male pheromone of sea lampreys comprises several minor components, including additional lamprey-specific, biologically active bile products released in smaller quantities by sexually mature males. Among these, 3,12-diketo-4,6-petromyzonene-24-sulfate (DkPES) is the most well-characterized. This bile alcohol has been shown to enhance the attractiveness of 3kPZS when the two compounds are combined, facilitating the localization and selection of potential mates by nearby females [139]. Notably, the male-typical ratio of 30:1 (3kPZS to DkPES) is more effective at attracting and retaining females in artificial nests than 3kPZS alone [136,139]. Both compounds are detected by distinct receptors in the MOE of adult sea lampreys; however, the detection threshold for DkPES is a thousandfold higher than that of 3kPZS [136]. This disparity in sensitivity suggests that DkPES may not be perceptible until the female is in proximity to males. Consequently, the authors hypothesized that DkPES serves as a cue for females to assess their distance from males [136].

#### 2.5.3. Other Bile Products

Compounds of the male pheromone necessary to maintain females in the nest for near-source courtship and spawning behavior also remain unidentified but may include other recently discovered bile products that stimulate the MOE and elicit behavioral responses. These lamprey-specific bile products extracted from sexually mature male washings are petromyzestrosterol [141], petromyzones A, B and C [142], petromyzenes A and B [143], and petromylidenes A, B, and C [144]. All of them are detected in the picomolar or sub-picomolar range by the female MOE, except for petromylidene A (10^−9^ M) and petromyzestrosterol (10^−6^ M). Moreover, these bile products induce behavioral responses in laboratory settings: all are attractive to females except for petromyzone B and C, which are repulsive, and petromyzestrosterol that remains untested. Another noteworthy bile product is 3-keto-1-ene petromyzonol sulfate, a lamprey-specific unsaturated sulfated bile alcohol which has the same potency as 3kPZS in attracting sexually mature females to nests [145]. However, a mixture of 3kPZS and 3-keto-1-ene petromyzonol sulfate shows no additive effects compared with 3kPZS alone, which suggests both molecules may bind to the same olfactory receptor and are therefore perceived as the same stimulus.

#### 2.5.4. Spermine

In the natural habitat, breeding pairs can engage in three-day sequences of nest maintenance and spawning [112]. However, no odorant mixtures can induce these behaviors except for the complete male odor. Indeed, the above-mentioned bile products are insufficient to retain females on nests for such extended durations, which suggests that other factors promote the maintenance of spawning pairs [76]. Remarkably, a molecule was found in sea lamprey semen that could act as a reliable and localized signal of nearby spawning males. Initially discovered in human seminal plasma in the 17th century [146], spermine is an odorous polyamine found in various organisms and tissues. Sea lamprey milt contains high levels of spermine, which promotes attraction specifically in sexually mature females [137,147,148]. Spermine stimulates the MOE at concentrations as low as 10^−14^ M, and interestingly, an olfactory receptor involved in its detection has been identified after screening most receptors expressed in the sea lamprey MOE [137]. This constitutes a male chemical signal that is detected by females with subpicomolar sensitivity and induces mating behaviors. Moreover, female sea lampreys prefer male-conditioned water when it is mixed with seminal plasma, and this behavioral bias increases with seminal plasma concentration [148]. Spermine is a reliable indicator of sperm availability and could also contribute to synchronized gamete-release, which is crucial for productive external fertilization. Furthermore, since milt itself is attractive to migratory and sexually mature sea lampreys of both sexes, additional yet unidentified components of the semen could draw males and migratory females to areas where active spawning occurs [137].

### 2.6. Section Summary

The olfactory system of lampreys is highly developed. It plays a crucial role throughout their life cycle, during behaviors such as predation, predator avoidance, migration, and reproduction, including mate selection, nesting, and spawning. While extensive research has explored the olfactory ecology of sea lampreys, efforts to identify individual compounds responsible for odor-mediated behaviors have only partially characterized the chemistry of lamprey pheromones and alarm cues [149]. Indeed, olfactory stimuli comprise complex mixtures of chemical components and the identities, concentrations, and ratios of these components in lamprey pheromones and alarm cues remain to be fully elucidated. For instance, mature male washings are significantly more attractive to mature females than any combination of individual compounds tested to date. Unfortunately, extracting lamprey-specific bile products in quantities sufficient for behavioral testing in natural streams poses a significant challenge [150]. Consequently, characterizing their behavioral effects both in isolation and in various mixtures will prove difficult, particularly in the natural habitat [151]. Furthermore, the chemical components found within sea lamprey pheromones induce distinct physiological and behavioral responses, and their signaling mechanisms are currently an active area of research. Ongoing studies aim to characterize the neuroendocrine and neuronal events that occur in response to odor detection in lampreys and are explored in the following sections.

## 3. Endocrine Signaling Induced by Pheromone Detection

Pheromones released by sexually mature male sea lampreys induce immediate behavioral responses in migrating and spawning conspecifics. Notably, these pheromones also trigger remarkable developmental and physiological effects in adult sea lampreys, such as gametogenesis [152] as well as the synthesis and release of pheromones in males [153,154]. Indeed, exposure to washings of sexually mature males accelerates sexual maturation in both immature males and females, leading to an earlier onset of spermiation and ovulation [152]. Additionally, once sexually mature, males upregulate the synthesis and release of pheromonal compounds [118,119,154] and further boost this production immediately upon exposure to 3kPZS [153]. Here, we first provide a comprehensive overview of the lamprey HPG axis and then describe the endocrine signaling mechanisms that alter the reproductive status and pheromone release patterns in sea lampreys following pheromone detection in their aquatic environment.

### 3.1. The Hypothalamic-Pituitary-Gonadal Axis in Lampreys

In lampreys, one of the oldest extant vertebrates, the primary regulator of reproductive physiology is the HPG axis (for reviews, see [155,156]), as in the rest of vertebrates [157]. The HPG axis, which bridges environmental inputs to reproductive outputs [158,159], has evolved early in the vertebrate lineage [160].

#### 3.1.1. Hypothalamic Gonadotropin-Releasing Hormones in Lampreys

In the hypothalamus of sea lampreys, neurons that produce gonadotropin-releasing hormones (GnRHs) generate three distinct types of lamprey GnRH (lGnRH) neuropeptides: lGnRH-I [161], lGnRH-II [162], and lGnRH-III [163]. These neuropeptides play a crucial role in the control of reproduction by regulating the pituitary-gonadal axis [164,165,166,167,168,169,170,171,172,173,174,175,176]; reviewed in [177]. Lamprey GnRHs bind to three distinct GnRH receptors (GnRH receptor 1–3 [178,179,180]; for a review, see [181]) found in both the pituitary gland [180,182,183] and the gonads [178,180,183,184].

In gnathostomes, GnRHs stimulate the pituitary gland, triggering the release of gonadotropins such as luteinizing hormone (LH) and follicle-stimulating hormone (FSH) into the bloodstream. These hormones then act on the gonads to promote the production of gametes and hormones. In contrast, a single functional gonadotropin, the lamprey glycoprotein hormone, was identified from the lamprey pituitary gland [185,186,187] and represents an ancestral type of gonadotropin absent in gnathostomes [156].

Moreover, a second pathway where lGnRHs directly stimulate gonadal tissues without acting on the pituitary gland may exist in sea lampreys [152]. Evidence supporting this hypothesis includes: (1) the presence of lGnRHs in plasma [152,188,189], (2) the detection of GnRH receptors in the testes [178,183,184] and ovaries [180,183,184], and (3) lGnRHs stimulating the release of hormones from in vitro-isolated lamprey gonads [184,190]. Thus, lGnRH peptides may be released into the bloodstream to act directly on the gonads, bypassing the pituitary gland [152]. Nevertheless, it is well-established that lGnRHs stimulate the pituitary-gonadal axis in adult sea lampreys (for reviews, see [155,156,160,191,192,193]) and that lGnRHs are biologically active in adult sea lampreys, inducing gonadal maturation [194], gonadal steroidogenesis [162,163,184,190,195,196,197,198,199,200,201,202], and gametogenesis [184,195,196,197,198,203]. Hence, as in other vertebrates, the HPG axis controls reproduction in lampreys.

#### 3.1.2. Gonadal Hormones in Lampreys

In lampreys, the roles of gonadal hormones are similar to those in gnathostomes [155]. As the output of the HPG axis, these hormones regulate reproductive functions throughout the body. In vertebrates, gonadal hormones exert both paracrine functions (i.e., promoting gametogenesis), and endocrine functions by being released into the bloodstream and binding to receptors in various target tissues to stimulate the development of secondary sexual characteristics. However, while research at the hypothalamic and pituitary levels has yielded conclusive findings (see Section 3.1.1), investigations into the identity and reproductive functions of gonadal hormones in lampreys have faced challenges. Like many other aquatic vertebrates [204,205], lampreys rely on gonadal hormones that significantly differ from those found in mammals [155]. Although the three major classes of vertebrate gonadal hormones, namely androgens (primary male sex hormones in mammals), estrogens, and progestogens (primary female sex hormones in mammals), are present in lampreys, the understanding of their biologically active forms remains limited.

The “classical” or “mammalian” main gonadal hormones, including testosterone, estradiol, and progesterone, have been identified in the plasma of lampreys [206], but among those, only estradiol is found in high concentrations [207,208,209,210], whereas testosterone and progesterone levels are either very low or undetectable [206,208,209,211,212,213,214]. In lampreys, “mammalian” gonadal hormones have been linked to key reproductive functions, such as gametogenesis (Androstenedione: [215]; Testosterone: [216]; Progesterone: [197,198,217]; Estradiol: [184,195,197,198,207,208,209,210,217,218,219,220,221,222,223]), spawning behavior (Estradiol: [209]), and the development of secondary sexual characteristics (Androstenedione: [215]; Testosterone: [224]; Estradiol: [217,224]; reviewed in [193]). However, the precise identity and role of biologically active gonadal hormones in lamprey reproduction remain unclear; reviewed in [155].

Growing evidence suggests that lamprey gonadal hormones differ structurally from those of other vertebrates due to the presence of an additional hydroxyl group at the C15 position. Specifically, testosterone and progesterone are converted in the gonads by 15-hydroxylase enzymes into their 15-hydroxylated derivatives [201,225,226,227,228] such as 15α-hydroxytestosterone [229] and 15α-hydroxyprogesterone (15α-P) [199]. Notably, these 15-hydroxylated steroids had never been identified as major metabolic products of the gonads in any other vertebrate [155]. While “classical” gonadal hormones were traditionally assumed to be the primary regulators of reproductive functions, it has been hypothesized that in lampreys, they serve instead as inactive precursors that are metabolized into biologically active hormones within the gonads [230].

Among these, 15α-P appears to be the primary ligand of progestogen receptors, rather than progesterone itself [231]. This conclusion is supported by four key findings: (1) progesterone is efficiently converted to 15α-P in the gonads [199,231]; (2) following lGnRH injections, plasma levels of 15α-P greatly exceed those of progesterone [190,198,199,202]; (3) 15α-P seems to be the most abundant gonadal hormone in sea lampreys [199,231,232,233]; and (4) lamprey progesterone receptors [234] possess a singular binding site that is unique among vertebrates and induces favorable, stabilizing interactions with the 15-hydroxyl group on 15α-P [235]. Moreover, both 15α-P and 15α-hydroxytestosterone have been associated with reproductive functions [152,199,202,217,227,229,231]. Thus, as observed in many other aquatic vertebrates [155,204,205], reproductive functions are predominantly regulated by “non-classical” gonadal hormones [155].

In vertebrates, including lampreys, the hypothalamus and pituitary gland play key roles in promoting the synthesis and release of gonadal hormones that regulate reproductive functions. Interestingly, exposure to pheromones can significantly influence these reproductive processes by activating the HPG axis and increasing gonadal steroidogenesis. The following subsection focuses on the endocrine signaling mechanisms through which pheromones stimulate gametogenesis [152] and pheromone production [153,154].

### 3.2. Exposure to Pheromones Induces Gametogenesis and Pheromone Production

Male sea lampreys migrate to spawning habitats and, upon reaching sexual maturity, release pheromones that strongly influence the behavior of conspecifics, triggering upstream movement and nesting/spawning activity [114]; see Section 2. Remarkably, beyond these behavioral effects, male pheromones also induce physiological changes, including an acceleration of gametogenesis in both sexes [152] and an increase in pheromone production in males [153,154]. The major component of the male pheromone, 3kPZS, plays an important role in these physiological responses by activating the HPG axis [152].

#### 3.2.1. Exposure to Pheromones Primes the Hypothalamic-Pituitary-Gonadal Axis

Exposure to 3kPZS in the aquatic environment stimulates the synthesis and release of lGnRHs [152]. In adults, this exposure leads to increased brain expression of the immediate early genes *Jun* and *JNK*, which are involved in the signaling cascades of mammalian GnRH receptors [236,237,238,239,240]. It also upregulates lGnRH-I and lGnRH-III gene expression, resulting in elevated levels of lGnRH-I and lGnRH-III peptides in the brain and plasma. Interestingly, 3kPZS exposure alters the output of the HPG axis as well, as evidenced by increased plasma levels of 15α-P [152,233]. This increase is likely driven by the enhanced lGnRH production, given that injections of lGnRH-I or lGnRH-III similarly induce a significant rise in 15α-P plasma levels in sea lampreys [199,202]. Hence, 3kPZS released by sexually mature males appears to (1) upregulate lGnRH gene expression in the brain, and (2) increase lGnRH peptide levels in both the brain and plasma, which (3) bypass the pituitary gland to act directly on the gonads, stimulating steroidogenesis, and ultimately (4) elevate 15α-P plasma levels [152].

#### 3.2.2. Gonadal Hormones Induce Gametogenesis and Pheromone Production

Exposure to gonadal hormones leads to the development of primary and secondary sexual characteristics in lampreys [215,224]. The biological functions of 15α-P have been studied [231] and this gonadal hormone contributes to regulating crucial physiological phenomena for male sea lamprey reproduction: gametogenesis and pheromone production. Notably, expression of progestogen receptors (nuclear progestin receptor and progesterone receptor membrane component) occurs in tissues that are presumed targets for gonadal hormone activity in males, including the testes, and the organs responsible for the production (liver) and release (gills) of pheromones [231]. Also, high-affinity binding of progestogen receptors is observed in both the nuclear and membrane fractions of target tissues (testes, liver, and gills), suggesting a widespread action of progestogen signaling [231]. Moreover, since the expression of progestogen receptors is higher in the testes, liver and gills of sexually mature males and increases in response to lGnRH injection [231], it appears that the actions of progestogens in male sea lampreys are relevant to sexual functions such as gametogenesis and pheromone production. Indeed, implanting immature males with time-release pellets of progesterone increases plasma levels of 15α-P and, remarkably, accelerates sexual maturation as the onset of spermatogenesis occurs sooner, most likely following the binding of 15α-P to progestogen receptors within the testes [231]. In addition, males implanted with progesterone exhibit a significant increase in plasma levels of 3kPZS, suggesting that progestogen signaling is also responsible for pheromone production. Activation of progestogen receptors by 15α-P in the liver and the gills may therefore induce the developmental changes necessary to sustain high pheromone production and release in male sea lampreys upon sexual maturation.

Male sea lampreys produce much higher pheromone levels than females, and these levels are significantly increased following sexual maturation [52,118,119,154]. Thus, physiological mechanisms that upregulate pheromone biosynthesis and release must occur in mature males. Indeed, analysis of metabolomes [119], anatomy [117], and gene expression [118] show that various mechanisms take place after sexual maturation to induce (1) synthesis of PZS as a precursor in the liver, (2) transportation via the bloodstream, and (3) uptake and modification to 3kPZS in the gills before (4) release into the environment. Foremost, it is worth noting that lamprey pheromones are bile products such as bile acids, salts and alcohols, which are lipid molecules synthesized primarily in the liver from cholesterol. Notably, lampreys produce C24 5α-bile acids that are considered the most ancestral biological bile acids and are synthesized almost exclusively by jawless fish [241]. Although these molecules are already produced in the liver during the larval stage [58], mature males radically rearrange their metabolic pathways to maximize the hepatic production of bile products [119]. Primarily, fat metabolism is adjusted by upregulating cholesterol-derived bile product biosynthesis while downregulating the biosynthesis of other lipids, such as fatty acids and leukotrienes. Also, amino acid metabolism is altered and biosynthesis of cofactors and vitamins is upregulated, whereas a downregulation in carbohydrate, energy, and Krebs cycle-related metabolisms occurs [119]. Interestingly, anatomical experiments targeting pheromones with immunocytochemistry revealed much more intense and widespread labeling of the liver in mature males than in immature males [117]. Notably, pheromone immunostaining is observed in hepatocytes with diffuse labeling of the cytoplasm and strongly labeled cytoplasmic granules.

Moreover, upon sexual maturation, the transcription of specific genes associated with pheromone production in the liver and active secretion into the bloodstream is substantially upregulated [118]. In the liver of mature males, three members of the cytochrome P450 monooxygenases with key roles in both classical and alternative pathways of bile acid synthesis are amplified in comparison with immature males. Namely, transcription of *CYP7a1* (cholesterol 7α-hydroxylase), which produces the initial and rate-limiting enzyme in the biosynthetic pathway of bile products from cholesterol, is increased 8000-fold, while *CYP27a1* (sterol 27-hydroxylase) and *CYP8b1* (sterol 12-α-hydroxylase), which both oxidize cholesterol intermediates, are also upregulated. Moreover, bile product efflux from hepatocytes is heightened by increasing expression of bile salt transporter Bsep, the main bile salt exporter in hepatocytes [242], and decreasing expression of sodium/bile acid cotransporter *SLC10A1* that is critical for bile salt uptake into hepatocytes [243]. In mature male sea lampreys, hepatocytes are thus involved in the upregulated biosynthesis of pheromonal bile products.

Hepatic bile products are generally excreted through the intestine during the larval stage [51,58] and then through the kidney in adults [244], since bile ducts degenerate during metamorphosis [116,245]. Moreover, to facilitate lipid digestion in juveniles following biliary atresia, de novo synthesis and secretion of taurine-conjugated bile products occur in the intestines [246]. Interestingly, in sexually mature males, specific mechanisms that allow the synthesis and excretion of pheromonal bile products through the gills exist [117,118,247]. Indeed, cytoplasmic granules were strongly labeled by antibodies directed against 3kPZS in subpopulations of gill epithelial cells in sexually mature males only [117]. Moreover, analysis with electron microscopy revealed ultrastructural characteristics in these cells that are compatible with lipid metabolism and storage of secretory products [117]. For example, prominent smooth endoplasmic reticulum, an organelle responsible for synthesizing steroid compounds, and widespread granules of varying electron density were observed. Accordingly, in the gills of sexually mature males, specific genes associated with the uptake of PZS from the bloodstream, modification to 3kPZS, and release into the water are upregulated [118]. Notably, *SLC10A2*, a sodium/bile acid cotransporter that has been shown to transport PZS with high affinity [248], is upregulated and may thus facilitate uptake of PZS from the plasma by gill epithelial cells [118]. The dehydrogenation of PZS to form 3kPZS may then occur via *HSD3B7* (3β-hydroxysteroid dehydrogenase 7), an enzyme that catalyzes this reaction [249,250] and is upregulated as well in male gill tissue [118]. Finally, transcription of enzymes that catalyze sulfation of bile products (*SULT2A1*; bile salt sulfotransferase) and other steroids (*SULT2B1*; hydroxysteroid sulfotransferase 2B1b) is also increased in gill epithelia [118], which may enable excretion of 3kPZS into the lumen by increasing its solubility [251]. Specialized cells of the gill epithelium may thus actively excrete 3kPZS. Interestingly, studies measuring the concentrations of PZS and 3kPZS in the liver, blood, gills, and holding water support the existence of a pheromone biosynthesis pathway originating in the liver where PZS is produced as a precursor, released in the plasma, and taken up in the gill epithelia where metabolization to 3kPZS occurs prior to discharge into the environment [118,153]. Altogether, the literature reviewed here shows that physiological changes in the liver and the gills enable the development of a biosynthetic pathway for the production and release of massive amounts of pheromones that is sex- and maturation-dependent, i.e., specifically in mature males.

#### 3.2.3. Hypothetic Signaling Pathway Inducing Physiological Effects in Response to Pheromone Exposure

**Figure 2:**
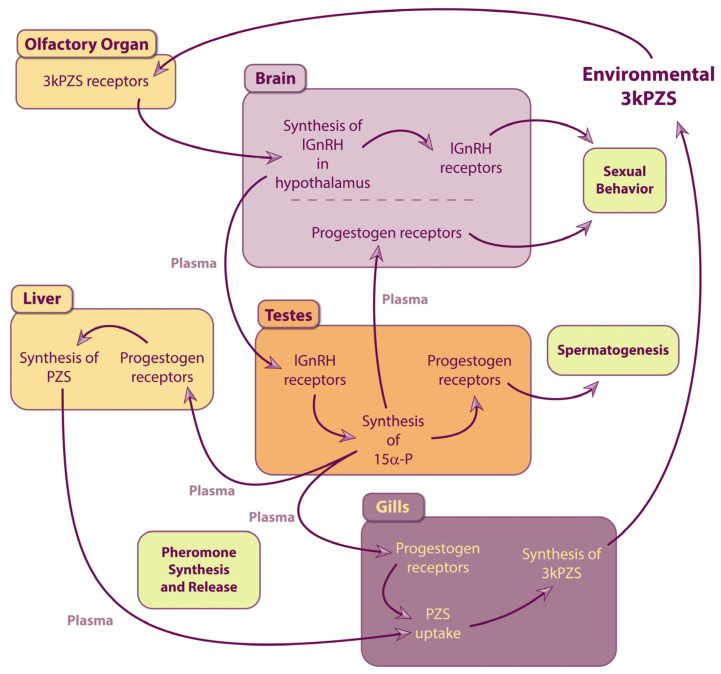
Neuroendocrine pathways induce physiological effects in male lampreys in response to pheromone detection. In male lampreys, the detection of a sex pheromone compound, 3-keto petromyzonol sulfate (3kPZS), by the olfactory organ leads to increased synthesis of lamprey gonadotropin-releasing hormone (lGnRH) and release into the plasma. In the testes, lGnRH activates receptors that induce steroidogenesis, including the synthesis of 15α-hydroxyprogesterone (15α-P). Locally, 15α-P then activates progestogen receptors that induce spermatogenesis. Moreover, 15α-P is also released in the plasma and travels to the liver to increase the synthesis and release of petromyzonol sulfate (PZS). In addition, 15α-P activates progestogen receptors in the gills, inducing uptake of PZS from the plasma and its conversion to 3kPZS, which is discharged into the environment. The increased 3kPZS levels in the environment may then stimulate other male lampreys to induce the same effects, allowing for synchronized sexual maturation and pheromone release following upstream migration to spawning areas. In addition, both lGnRH and 15α-P may activate receptors within the brain to promote sexual behavior. This neuroendocrine pathway illustrates how pheromone inputs may induce important physiological effects in male lampreys.

In sea lampreys, pheromones emitted by sexually mature males exert significant physiological actions in receivers, including an acceleration of gametogenesis [152] and an increase in pheromone release specifically in mature males [153,154]. Although further examination is needed to achieve a broader understanding, the mechanisms that mediate these physiological effects are partly characterized. Here, we propose a signaling pathway (Figure 2) to describe how the detection of male pheromones can induce these physiological responses: (1) chemosensory detection of 3kPZS (2) recruits the HPG axis and induces upregulation of hypothalamic lGnRH expression that leads to (3) an increase in steroidogenesis with elevated 15α-P synthesis in the gonads [152]; (4) 15α-P may then activate progestogen receptors located (4.1) in the gonads to induce gametogenesis, and (4.2) in the male liver and gills to upregulate pheromone biosynthesis and release [231].

Evidence suggests that 15α-P plays a central role in mediating the downstream effects of 3kPZS exposure. Male pheromone exposure increases 15α-P levels [152], and treatments with either male pheromones or 15α-P induce both spermatogenesis [152,231] and pheromone production [153,154,231]. Since spermatogenesis is regulated by the HPG axis [191] and coincides with pheromone release [52], it was hypothesized that both processes are governed by the HPG axis [252]. More recently, researchers proposed that spermatogenesis and pheromone release were induced by the same gonadal hormone: 15α-P [155,231], because both begin when 15α-P levels peak and progestogen receptor expression increases in the mature male testes, liver, and gills [231].

Research into the roles of additional pheromone components, hypothalamic hormones, and gonadal hormones is warranted to improve our knowledge about the regulation of the reproductive system following pheromone detection. In sea lampreys, 3kPZS not only alters the levels of lGnRHs and 15α-P [152,233], but also the concentrations of other hypothalamic hormones such as the gonadotropin-inhibitory hormones (GnIHs) [189,233], and other gonadal hormones including testosterone, androstenedione, progesterone, and estradiol [233]. Gonadotropin-inhibitory hormones regulate reproduction by modulating GnRH functions [253,254] and were identified in sea lampreys [255,256]. Moreover, as reviewed above (Section 3.1.2), gonadal hormones other than 15α-P have been associated with gametogenesis, the development of secondary sexual characteristics, and spawning behavior in sea lampreys. Furthermore, the levels of GnRHs, GnIHs and gonadal hormones are altered differentially in response to 3kPZS depending on the sex and maturity status of the animals [152,233]. To further complicate matters, other components of the male pheromone may similarly modulate the HPG axis. For example, 3-keto allocholic acid [249], which is a male bile acid structurally similar to 3kPZS and also detected by the MOE [125], but devoid of known behavioral effects [114,138], also alters the levels of lGnRHs [188], GnIHs [189,233], and gonadal hormones [188,233] in a sex- and maturity-dependent fashion. Importantly, exposure to 3kPZS or 3-keto allocholic acid produces distinct effects on gametogenesis [152,188], gonadal steroidogenesis [233], and pheromone production [154]. Therefore, the regulation of reproduction induced by lamprey pheromones is a multifaceted phenomenon that deserves further examination.

#### 3.2.4. Impacts on Reproduction

In sea lampreys, the spawning season lasts approximately 2–3 months, during which migratory adults with variable reproductive statuses reach the spawning grounds at different times [112]. In this sexual selection context, males benefit from releasing pheromones into the environment. Since sexual maturation occurs in males during upstream migration, pheromone excretion is massively increased upon reaching spawning grounds. As seen in Section 2, male pheromones induce behavioral responses in females that are attracted upstream from long distances to spawning sites and then drawn and retained into nests for mating [113]. Additionally, this pheromonal signal produces physiological responses in both males and females by accelerating gametogenesis [152]. This phenomenon elicits reproductive synchrony so that males and females become sexually mature at the same time and place, which is highly beneficial because lampreys die shortly after reaching sexual maturity.

Furthermore, male pheromones increase pheromone production and release in other sexually mature males [153,154,231]. Indeed, an increase in 3kPZS discharge happens in minutes after exposure to 3kPZS [153,154]. Moreover, 3kPZS exposure also upregulates the excretion of lesser amounts of other bile products (PZS, 3-keto allocholic acid, and allocholic acid) in the environment by sexually mature males [154]. Several reproductive benefits may result from increasing pheromone release in response to pheromone detection. For example, synchronous pheromone discharge from males occupying a specific spawning site will produce a collective, aggregate pheromone signal that should result in higher pheromone levels available downstream for migrating females. This signaling cooperation should consequently increase the number of migrating females attracted to their spawning area and/or hasten their arrival, thus raising the probability of reproductive success for all males in the area. In contrast, in the spawning lek, signaling competition occurs between nesting males since females choose nests with higher concentrations of 3kPZS [113], and a high variability of 3kPZS production exists between different males [121]. Thus, since 3kPZS contributes importantly to nest localization, nest selection, and mate choice of mature females [113,114,129], individual males should benefit from maximizing pheromone excretion [153], which enhances mating competitiveness and the probability of retaining a female in the nest for spawning. Therefore, amplifying pheromone release in response to pheromone detection should increase male reproductive success.

### 3.3. Section Summary

Pheromonal bile products play a crucial role throughout the life cycle of sea lampreys, especially for reproduction. They have evolved signaling mechanisms to synchronize sexual maturation and facilitate the timely congregation of individuals in spawning grounds. The characterization of the lamprey HPG axis and the conspecific chemosensory cues that activate it has provided new insights into the signaling mechanisms through which male pheromonal compounds stimulate gametogenesis in sexually immature adults [152] and trigger the immediate release of 3kPZS in sexually mature males [153,154].

In this section, we propose a pathway in which pheromonal stimulation of the HPG axis leads to the production of gonadal hormones that induce developmental changes in the testes, liver, and gills, thereby accelerating spermatogenesis and enhancing pheromone production. Nevertheless, the male sea lamprey odor comprises multiple components, each with distinct functions, necessitating further investigation into the roles of various pheromonal compounds and their effects on the HPG axis, gonadal hormones, and, ultimately, reproductive functions. Additionally, neuronal signaling mechanisms and pathways that influence behavior following odor detection have been observed within the lamprey central nervous system and are explored in the next section.

## 4. Neuronal Mechanisms Induced by Odor Detection

In Section 2, we explored how olfactory stimuli impact lamprey behavior. As Nikolaas Tinbergen, a founding father of ethology, famously noted [257], understanding behavior requires exploring its mechanisms (or causation)—specifically, the physiological processes driving behavioral responses. Indeed, to comprehend animal behavior, one must study what stands between stimuli and responses, which is the nervous system. In lampreys, olfactory behavior hinges on neuronal activity within the nervous system that detects chemicals in the environment and then triggers intricate patterns of muscle contractions (referred to as behavioral responses). Moreover, these responses are adapted to environmental conditions and the animal’s internal state. Given that behavior fundamentally stems from cellular activity within the nervous system, analyzing behavior must include the causative neuronal mechanisms. Therefore, this section explores the neuronal signaling pathways that link olfaction to behavior in lampreys.

### 4.1. Olfactomotor Circuitry in Lampreys

The lamprey brain is considered an ancestral vertebrate brain, serving as a blueprint for the mammalian brain and sharing similarities in basic organization and neuronal properties; for reviews, see [258,259,260,261,262,263,264,265,266]. Indeed, the central nervous system of lampreys contains all major subdivisions found in vertebrates (telencephalon, diencephalon, mesencephalon, rhombencephalon, and spinal cord), and most of its main neuroanatomical structures can be homologized with those of gnathostomes [267], albeit in a less complex form.

Moreover, the lamprey olfactory system is well characterized, and its anatomy suggests a strong reliance on odor detection for guiding behavior. The nasal cavity, which houses the olfactory epithelia, outsizes the entire brain [268]. The olfactory bulbs (OBs), responsible for processing olfactory inputs, are notably large compared to other vertebrates [269], even surpassing the size of the cerebral hemispheres [270]. These structural adaptations allow lampreys to detect soluble molecules in their aquatic environment with remarkable sensitivity (see Section 2).

Odorants induce behavior by stimulating the central nervous system, which then generates an appropriate behavioral response. In lampreys, the neuronal signaling pathway, from odor detection in the peripheral olfactory organ, through the brain, to the spinal motoneurons that control muscle contractions and movement, has been extensively studied; for a comprehensive review, see [271]. Notably, the sea lamprey is currently the only vertebrate in which this signaling pathway has been identified [272]. Within the nasal cavity, olfactory sensory neurons detect chemosensory cues from the environment and relay signals to the OB. These signals are then processed in several brain regions, including the posterior tuberculum (PT), the mesencephalic locomotor region (MLR), and the reticulospinal (RS) neurons, before reaching the locomotor central pattern generators in the spinal cord, which generate rhythmic swimming patterns. The neuronal circuitry described in this section plays a crucial role in transforming olfactory inputs into locomotor responses.

#### 4.1.1. Olfactory Organs and Receptors

The nasal cavity of lampreys contains two distinct olfactory organs interconnected by minute ducts: the MOE and the accessory olfactory organ (AOO) [273,274,275,276]. Both organs feature chemosensory epithelia composed of olfactory sensory neurons that detect soluble substances and transmit olfactory signals to the brain. While electro-olfactography studies have identified various stimulatory substances for the MOE, experimental access to the AOO for electrophysiological recordings remains limited, hindering a comprehensive understanding of its chemosensory activity. The AOO of lampreys offers an interesting comparison to the vomeronasal organ of tetrapods [277]. In mammals, the traditional view distinguishes the functional roles of the main olfactory system, which detects conventional odorants, and the vomeronasal system, which specializes in detecting pheromones. Today, it is well established that both systems participate in pheromone detection [278,279]. However, the specific contributions of the MOE and the AOO to chemosensory-induced behavior in lampreys remain unknown.

Olfactory signaling pathways are initiated when odorants bind to olfactory receptors in the nasal cavity. Gene families that encode a diverse repertoire of olfactory receptor subtypes enable the detection and discrimination of numerous odorants. In lampreys [280,281], as in the rest of vertebrates, olfactory sensory neurons express a single receptor subtype and are broadly distributed within the olfactory epithelium. Their olfactory receptors share characteristic structural hallmarks with those of other vertebrates, reflecting their ancient evolutionary origins [281].

The four main families of olfactory receptors in vertebrates have been identified in the sea lamprey genome, including 40 odorant receptor (OR) genes [282,283], 33 trace amine-associated receptor-like (TAAR) genes [282,284,285,286], 6 vomeronasal type 1 receptor (V1R) genes [282,287,288], and a single vomeronasal type 2 receptor (V2R) gene [288]. Moreover, all these receptor families are expressed in the peripheral olfactory organ of lampreys [137,276,280,281,282,283,286,287,288], with the exception of V2Rs, whose olfactory function has not been demonstrated yet [288]. Also, lamprey TAARs are considered as “ancestral” receptors that emerged specifically in lampreys, as they lack the conserved TAAR signature motif that appeared in jawed vertebrates [282,284,285,286].

Ligands have been identified for several lamprey olfactory receptors, such as compounds from sea lamprey pheromones that induce migration and nest attraction behaviors [283]. Two ORs, OR320a and OR320b, activate olfactory sensory neurons upon exposure to 3kPZS. Multiple other bile products also bind to these receptors, including potent agonists (PZS and others), partial agonists (DkPES and others), and allosteric inhibitors (allocholic acid). Moreover, spermine, a sex pheromone compound found in the sea lamprey seminal fluid, is detected by both TAAR348 [137] and TAAR365 [147]. Interestingly, cadaverine and putrescine, polyamines hypothesized to be repellents for sea lampreys [89], activate TAARs like TAAR346a [137,286] and TAAR365 [147]. Other TAARs were also de-orphanized [286], such as TARLL1b (bound by indole and its derivatives), TARLL3h (bound by a few diamines), and TARLL4a (broad range of ligands including primary and secondary amines). Lastly, no ligands are currently identified for lamprey V1Rs and V2Rs.

In comparison with the MOE, the expression profile of olfactory receptors in the AOO has been less explored. Initial studies suggested that ORs, TAARs, and V1Rs are expressed in the AOO, and that its expression profile is virtually identical to that of the MOE [276]. However, recent findings indicate the absence of V1Rs in the AOO [288], necessitating further investigation to delineate the specific roles of both olfactory organs in lamprey olfactory-induced behaviors.

#### 4.1.2. Olfactory Bulb Circuitry

From the nasal cavity, olfactory sensory neurons transmit signals to the brain via their axons, which collectively form the olfactory nerve. Three distinct morphotypes of olfactory sensory neurons have been identified in the MOE of sea lampreys: short, intermediate, and tall [289]. In contrast, the AOO contains only short, cuboidal olfactory sensory neurons [275]. These neurons give rise to two separate olfactory pathways: projections from the MOE extend to the main OB (MOB), while those from the AOO terminate in the medial OB (medOB) [275,276,290]. Within the OB, axons of olfactory sensory neurons synapse onto projection neurons, which relay olfactory signals to higher brain centers for further processing [291]. The medOB and MOB contain two spatially segregated and anatomically distinct populations of projection neurons [292]. The medOB houses a single large glomerulus that receives input exclusively from the AOO and is activated by amino acids, lamprey pheromones, and taurocholic acid [290]. In parallel, the MOB contains 41 to 65 smaller, spatially segregated glomeruli [293] that process inputs exclusively from the MOE [290]. Notably, odor response profiles differ between MOB subregions: the lateral MOB responds selectively to amino acids, whereas the dorsal MOB is preferentially activated by lamprey pheromones and taurocholic acid [290].

Thus, olfactory information is processed by two parallel pathways: the medial pathway (AOO → medOB) and the lateral pathway (MOE → MOB). The roles of each pathway in olfactory behavior are currently unknown, but it was proposed that they are involved in different functions [272,290,292,294], similarly to segregated olfactory subsystems observed in fish ([295,296,297,298,299,300,301,302,303,304,305,306,307,308,309,310,311,312,313,314,315,316]; reviewed in [317,318,319,320]), terrestrial vertebrates ([321,322,323,324]; reviewed in [317,325,326,327,328,329,330]), and even invertebrates ([331,332,333]; reviewed in [334,335]).

#### 4.1.3. Projections to the Posterior Tuberculum

In lampreys, OB projection neurons relay sensory signals to the PT, a key region involved in locomotor control. The PT integrates inputs from multiple sensory modalities [270,336], including olfactory [337], visual, and electro-sensory signals [338]. This region also contains dopaminergic neurons that project to various motor centers in the brain [270,336,339] and are considered the lamprey homolog of the substantia nigra pars compacta [339,340,341,342]. The PT plays a pivotal role in locomotor control, as evidenced by several findings: (1) its electrical or pharmacological activation induces swimming behavior [272,343,344,345,346], (2) dopaminergic neuron degeneration reduces both spontaneous and olfactory-induced locomotion [347], and (3) the PT is consistently active during swimming movements [337].

In lampreys, the OB projects directly to the PT [270,272,291,292,294,336,337,348,349,350,351]. Specifically, axons from both the medOB [337] and the MOB [291] extend to the PT, where they lie in proximity to its dopaminergic neurons. However, only medOB stimulation directly activates PT neurons, whereas MOB stimulation does not [337]. Instead, the MOB projects to the lateral pallium (LPal) [270,272,291,294,348,349,350,352], which in turn projects to the PT [294,336,353]. Thus, MOB activation recruits the PT indirectly through the LPal [294,336,337,353]. Notably, LPal projections are also positioned near PT dopaminergic neurons [336,353], and PT neurons are activated by LPal stimulation [337]. The LPal is a three-layered cortex that is considered as a precursor to the mammalian neocortex ([270,291,352,353,354,355,356,357]; reviewed in [263]). It includes a ventral region with olfactory functions [291] that has been proposed as the evolutionary precursor of the mammalian piriform cortex [348,350,355].

In summary, OB projection neurons convey olfactory signals to the PT via two parallel pathways: the medial pathway (medOB → PT) and the lateral pathway (MOB → LPal → PT). Interestingly, individual PT neurons integrate inputs from both the medOB and LPal, suggesting the existence of a common descending olfactomotor pathway where olfactory inputs from both the AOO and the MOE converge to trigger locomotion [337].

#### 4.1.4. Common Descending Locomotor Pathway

To induce swimming in lampreys, the PT recruits the MLR [344,345], a brainstem area essential for the initiation, maintenance, and termination of locomotion in lampreys ([358,359]; for reviews, see [360,361,362,363,364]) and other vertebrates [365]. In sea lampreys, the PT regulates MLR activity through glutamatergic excitation, which incrementally modulates swimming speed, while dopaminergic transmission provides additional excitatory input via D1 receptors [345]. These projections originate from PT neurons containing dopamine [337,344], glutamate, or both [345].

The MLR, in turn, recruits RS neurons to elicit swimming [358] through glutamatergic [366] and cholinergic [367,368,369] transmission. Additionally, RS neurons receive direct dopaminergic input from the PT, further enhancing their excitability [346]. In all vertebrates from lampreys to mammals [370,371], once activated, RS neurons stimulate the central pattern generators in the spinal cord [372,373], which coordinate rhythmic muscle contractions via motoneurons to produce locomotor movements; reviewed in [374]. The strength of transmission from RS neurons to the spinal cord determines swimming speed [366,375].

The olfactomotor pathways in lampreys provide a robust neural connection between olfactory sensory input and motor output. Indeed, exposure to specific odorants, such as L-arginine, 3kPZS, 3-keto allocholic acid, and taurocholic acid, elicits RS neuron activation [272]. Furthermore, electrical or pharmacological stimulation at multiple levels of these pathways—including the olfactory nerve [272,294,337,351,376,377], the medOB [272,294,337], the MOB [294], the LPal [294,337,353], and the PT [272,343,344,345,346]—triggers RS neuron activation and/or swimming. Thus, the medial and lateral olfactomotor pathways relay olfactory stimuli to motor regions in the brainstem, ultimately driving locomotion.

**Figure 3:**
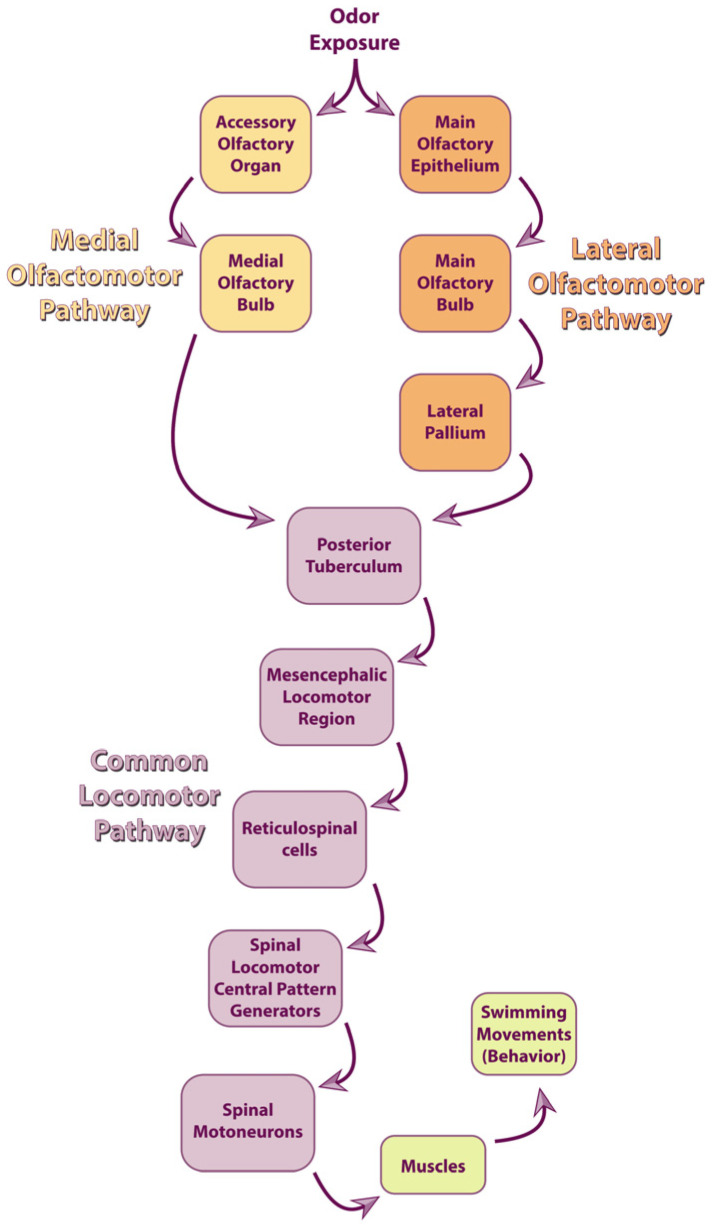
Olfactomotor pathways in the sea lamprey central nervous system transform odor input into motor output. Chemosensory cues in the environment enter the nasal cavity and reach the peripheral olfactory organ. In the medial olfactomotor pathway (yellow), sensory neurons of the accessory olfactory organ are activated by chemical cues and transmit the signal to the medial olfactory bulb, which then sends excitatory projections to the posterior tuberculum. In the lateral olfactomotor pathway (orange), sensory neurons of the main olfactory epithelium are activated by chemical cues and transmit the signal to the main olfactory bulb. In turn, the main olfactory bulb recruits the lateral pallium that sends excitatory projections to the posterior tuberculum. In the common locomotor pathway (pink), neurons of the posterior tuberculum target the mesencephalic locomotor region that controls the activity of reticulospinal cells. Excitatory projections then activate locomotor central pattern generators in the spinal cord that coordinate muscle contractions to produce swimming movements. This neuronal pathway illustrates how odor inputs are transformed into motor output by the sea lamprey central nervous system.

In summary, we describe two parallel olfactomotor pathways—originating from distinct sensory epithelia, the MOE and AOO—that regulate brainstem motor circuits responsible for locomotion (Figure 3). The medial pathway (AOO → medOB → PT) and the lateral pathway (MOE → MOB → LPal → PT) converge at the PT, which plays a pivotal role in controlling a common descending locomotor circuit (MLR → RS neurons → spinal cord → motoneurons → muscles). This network enables lampreys to navigate their environment in response to olfactory cues associated with foraging, predator avoidance, and mating. By transforming odor detection into locomotor responses, these neural pathways provide a mechanistic framework underlying the natural behaviors observed in response to environmental chemical signals, such as food-related odors, alarm cues, and pheromones.

### 4.2. Modulation of the Olfactomotor Circuitry

Significant progress has been achieved in identifying specific olfactory ligands that trigger stereotypical behaviors in various animals, including lampreys. However, the mechanisms enabling variability in olfactory-induced behaviors remain elusive [378]. In Section 4.1, we outlined the neural pathways responsible for innate, hardwired motor responses to olfactory stimuli, such as pheromones or food cues [272,294,337]. Yet, this olfactomotor circuitry does not account for the full spectrum of odor-driven behaviors observed in lampreys, which undergo variations throughout the life cycle and must adapt to diverse external (environmental) and internal (biological state and requirements) conditions.

This variability is termed behavioral plasticity, denoting adaptive changes in animal behavior in response to shifts in external or internal environments. In lampreys, responses to odor cues are significantly influenced by external factors such as the light-dark cycle, as well as internal factors like the animal’s sex and developmental stage. Therefore, modulation within the olfactomotor circuitry is essential to ensure that olfactory behaviors are adapted to both environmental contexts and internal needs. Here, we explore potential modulatory mechanisms within the olfactomotor pathways that could account for the plasticity of behavioral responses following odor detection.

#### 4.2.1. Neuromodulation in the Olfactory System

Modulation of the olfactory signal can provide strong control over the hardwired olfactomotor circuits that produce motor behavior in response to olfactory cues. Multiple mechanisms in the peripheral olfactory organ and the OB could adjust the olfactory signal before reaching downstream motor circuits. For instance, variations in olfactory receptor expression within the MOE and the AOO may contribute to sex-specific differences in olfactory-induced behaviors in lampreys. Indeed, sexually dimorphic expression patterns of olfactory receptors and related genes have been documented in the sea lamprey peripheral olfactory organ [276], as also observed in zebrafish [379]. While the analysis of olfactory receptor expression remains incomplete, distinct expression profiles of OR, TAAR, and V1R genes in adult males and females suggest a direct role in regulating olfactory behavior [276].

Beyond modulation in the peripheral olfactory organ, synaptic mechanisms within the OB can regulate olfactory input entering the brain. The OB of lampreys harbors multiple neuromodulatory systems, including γ-aminobutyric acid (GABA), dopamine, and serotonin. GABAergic modulation, provided by local inhibitory neurons in the OB [294,380], suppresses olfactory signal transmission from both the medOB and MOB to downstream motor circuits via GABA_A_ receptors [294,351].

Dopaminergic modulation in the OB is mediated by local dopaminergic neurons [336,339,340,351,381,382,383,384,385,386,387], while additional dopaminergic input to the medOB arises from neurons in the PT [351]. Within the medOB, D2 receptors are present [336], and their activation decreases RS neuron responses to olfactory input [351]. In contrast, both D1 and D2 receptors are present in the MOB [336,388,389], although their precise roles in sensory processing remain unclear. This suggests that dopaminergic modulation may regulate odor-driven behaviors through distinct mechanisms within the medial and lateral olfactomotor pathways.

Unlike dopaminergic and GABAergic neurons, serotoninergic neurons are absent from the OB but provide extrinsic modulation via projections from the peripheral olfactory organ [293,339,390,391,392] and likely from the mesencephalic tegmentum [339,390,393]. Serotonin suppresses MOB activity in response to various olfactory stimuli, including lamprey pheromones (3kPZS, PZS, 3-keto allocholic acid, petromyzonamine disulfate, and petromyzosterol disulfate), amino acid mixtures (L-arginine and L-histidine), and taurocholic acid [394]. This inhibition is mediated by 5-HT1a receptors [394], which are widely expressed in the OB [395,396].

In summary, sex-specific differences in olfactory receptor expression at the peripheral level may contribute to variability in olfactory-induced behavior. Meanwhile, at least three neurotransmitter systems—GABA, dopamine, and serotonin—modulate neural activity in the OB, providing additional layers of regulation that may underlie behavioral plasticity.

#### 4.2.2. External (Environmental) Signals Modulate Olfactory Behavior

Animal behavior is profoundly shaped by environmental context. For instance, sea lampreys are nocturnal creatures that remain mostly immobile during the day, suppressing olfactory-driven behaviors until nightfall. Predominantly nocturnal activity is observed throughout the lamprey life cycle, including the larval stage [7,397,398,399], downstream migration [11,111,400,401,402,403,404], foraging in lakes [405], stream searching [42], and upstream migration [1,2,42,97,111,406,407,408,409,410]. A notable exception occurs during spawning, when lampreys exhibit both diurnal and nocturnal activity [111,112,411]. During this period, sexually mature females increase their swimming activity in response to pheromone exposure even in daylight [114,122,123,129]. Nocturnality likely provides an adaptive advantage, as darkness reduces predation risk [122,399,412,413]. Consequently, olfactory-driven behaviors occur primarily at night during most of the life cycle.

How does light regulate odor-driven behavior? Studies on artificially blinded sea lampreys indicate that light avoidance is not vision-dependent [2], suggesting an alternative mechanism—possibly involving the pineal complex. This photosensory organ, located beneath a translucent skin zone [414,415,416,417], regulates circadian locomotor control [418,419]. Interestingly, interactions between the pineal complex and the olfactomotor circuitry have been proposed as a pathway for photic modulation of olfactory behavior [122,123]. Presumably, since pinealofugal projections terminate within the nucleus of the PT, and the mesencephalic tegmentum [420,421], inhibition of locomotor output may occur by suppressing activity in the PT, the MLR, and mesencephalic RS neurons, thereby overriding olfactory-driven movement during daytime despite exposure to attractive or repulsive odors.

Another mechanism for light-induced suppression of locomotion has also been suggested [2]. Light avoidance in lampreys may be mediated by dermal photoreceptors in the tail, which are linked to the lateral line system [422,423,424]. Indeed, illumination of these photoreceptors triggers burrowing in larvae [422,425] and refuge-seeking behavior in adults [2,423,424,426]. Photic input from tail photoreceptors is transmitted via the lateral line nerve to the medial octavolateral nucleus, which then influences RS neurons [424], potentially overriding olfactomotor signals in favor of refuge-seeking behaviors. Beyond light, other environmental factors modulate olfactory-driven locomotion. During upstream migration, movement is affected by water temperature [62,97,111,426,427,428], water acidity [429], and hydrological conditions [49,428,430].

In summary, lamprey olfactory behavior is intricately shaped by environmental cues, particularly light, which may suppress odor-driven locomotion through multiple neural pathways. However, the precise neuronal circuits underlying these modulatory effects remain largely unknown and warrant further investigation.

#### 4.2.3. Internal (Hormonal) Signals Modulate Olfactory Behavior

Behavioral output must be finely tuned to biological needs. As discussed in Section 2, sea lamprey reproductive behavior heavily relies on olfaction and differs significantly between sexes and developmental stages. Following sexual maturation, females exhibit distinct behavioral responses to male pheromones, in stark contrast to those of males and sexually immature females [52,60,63,115,123,124,129]. Such behavioral plasticity suggests the existence of modulatory mechanisms along the olfactomotor pathways, likely influenced by hypothalamic and gonadal hormones controlling olfactory behavior through physiological effects in the brain.

Hormone levels fluctuate in response to physiological states, serving as internal signals that convey information, such as sex and developmental stage, to neural circuits. These signals, in turn, modulate brain activity according to the reproductive state of the animal [431]. Notably, hormones such as GnRH and gonadal steroids serve as signals that depend on the reproductive state and can exert profound effects on olfactory processing. In fish, fluctuations in GnRHs, gonadal steroids, and their receptors within the olfactory system are closely tied to sex and reproductive condition, offering a potential mechanism for inducing plasticity in olfactory responsiveness and behavior [432,433,434,435,436,437]. Therefore, lGnRH and gonadal hormones are likely candidates for neuromodulation of the olfactomotor circuitry, aligning olfactory-driven behaviors with sex and developmental status in lampreys (Figure 2). In the following section, we explore potential modulatory mechanisms at the interface between the reproductive system and the olfactomotor circuitry that may underlie behavioral plasticity in response to odor detection.

##### Modulation of Olfactory Behavior by Gonadotropin-Releasing Hormones

Gonadotropin-releasing hormones are the primary regulators of the reproductive axis and may also modulate the olfactomotor pathways in lampreys, depending on sex and developmental stage. While GnRH stimulates the release of gonadotropins from the pituitary gland, it also plays a broader physiological role beyond the reproductive HPG axis. Specifically, non-hypophysiotropic GnRH acts as a neuromodulator within the vertebrate brain and olfactory epithelium, influencing neuronal excitability to regulate specific behaviors independently of pituitary function (reviews in fish and amphibians: [431,438,439,440,441,442,443]; reviews in mammals: [444,445,446]).

In fish and amphibians, non-hypophysiotropic GnRH modulates the excitability of olfactory sensory neurons [447,448] and OB projection neurons [436,449], suggesting a critical role in regulating olfactory responsiveness and behavior; for reviews, see [159,450]. In sea lampreys, lGnRH brain levels are sexually dimorphic [152,203] and vary significantly across developmental stages [167,172,174,194,203,210,221,451,452]. The complexity of lGnRH transmission in sea lampreys is further highlighted by the presence of three distinct ligands (lGnRHs I–III), which can be co-expressed within individual neurons [172,174,176,453]. Interestingly, the differential expression of these lGnRHs in the brain during development and sexual maturation suggests distinct physiological roles for lGnRH-I, -II, and -III in males and females at different life stages [156,176,203]. Since lGnRHs are found along the olfactomotor pathways, their release may contribute to the pronounced sex differences in olfactory-driven behaviors (Figure 2). Notably, lGnRH gene expression and fibers have been detected in several brain regions involved in sensory processing and motor control, including the OB [162,171,172,173,174,175,176], PT [172,454], mesencephalon [171,172,173,174,454] and rhombencephalon [162,176]. Further evidence for the role of lGnRHs in modulating olfactory behavior stems from studies showing variability in GnRH receptor expression between developmental stages and sexes in both the OB and mesencephalon [183]. Indeed, out of the three known GnRH receptors in sea lampreys ([178,179,180]; for a review, see [181]), only GnRH receptor 1 is present in the brain during the larval stage, whereas both GnRH receptors 2 and 3 are also expressed in juveniles and adults [183]. Interestingly, in the OB of adults, all three GnRH receptors are expressed, but with sex-specific differences—GnRH receptor 3 is absent in males but present in females [183]. Similarly, in the mesencephalon of adults, which houses the MLR, GnRH receptor 1 is exclusive to males, GnRH receptor 2 is found in both sexes but in distinct locations, and GnRH receptor 3 is absent [183]. While it remains unclear whether these receptors are expressed within the MLR itself, their differential distribution suggests that non-hypophysiotropic lGnRHs may regulate olfactory behavior through neuromodulatory effects in the OB, PT, and mesencephalon.

The significant developmental and sex-related differences in lGnRH distribution [176] and receptor expression [183] suggest that lGnRH signaling plays a key role in shaping the olfactomotor circuitry, driving behavioral plasticity in a context-dependent manner. However, direct physiological evidence of lGnRH neuromodulation in the lamprey brain remains elusive, and its broader roles beyond the HPG axis are still poorly understood. Investigating how lGnRH influences olfactory processing and behavior could provide crucial insights into the mechanisms underlying sexual dimorphisms in lamprey reproductive behavior.

##### Modulation of Olfactory Behavior by Gonadal Hormones

Gonadal hormones such as androgens, estrogens, and progestogens exert a profound influence on brain function and may also contribute to behavioral plasticity in lampreys. While their negative feedback on the hypothalamic-pituitary axis is well-established in mammals [455,456,457,458,459,460], these hormones also modulate neuronal excitability in brain regions beyond this axis. In vertebrates, sexually dimorphic reproductive behaviors are orchestrated in part by gonadal hormones acting on steroid-sensitive neural circuits; reviewed in [431,461,462,463,464,465,466,467,468,469].

Since olfactory behavior differs between male and female adult sea lampreys (see Section 2), sexual dimorphisms likely exist in their brains. This dimorphism could be the result of hormonal input, as in other vertebrates, in which behavior varies according to gonadal hormone levels and receptor expression. Indeed, plasma levels of gonadal hormones fluctuate widely in adult lampreys, differing between sexes and reproductive stages (reviewed in [155]), including estradiol [207,208,209,210,217,219,223,227,233], progesterone [209,210,217], 15α-P [152,199,202,233], testosterone [207,209,233], 15α-hydroxytestosterone [202,217,227,229], and androstenedione [227]. Notably, stereotypical spawning behaviors have been linked to changes in estradiol levels [209]. Moreover, multiple gonadal hormone receptors have been identified in the sea lamprey brain, including an androstenedione receptor [215], progesterone receptors [231], and an estrogen receptor [470]. The brain levels of these receptors differ between sexes [215] and reproductive stages [231]. Interestingly, in sexually immature female sea lampreys, in situ hybridization revealed the expression of estrogen receptors in the OB, mesencephalon, and rhombencephalon [470]. Additionally, earlier autoradiographic studies in sexually immature adult silver lampreys [169] and larval sea lampreys [170] identified estrogen target cells in a region corresponding to the rostral part of the dopaminergic nucleus of the PT [337,438], which was then identified as the central hypothalamus. Although the full distribution of gonadal hormone target cells in the lamprey brain remains unclear, the presence of estrogen receptor sites in the OB, PT, mesencephalon, and rhombencephalon suggests that gonadal hormones influence neuronal activity within the olfactomotor pathways. Sensitivity to gonadal hormones in this circuitry could induce plasticity in olfactory behavior (Figure 2). For instance, estrogens may exert sexually dimorphic modulatory effects on odor processing in the OB and modulate neural responses to olfactory inputs in the PT, MLR, and rhombencephalic RS neurons [159,431].

Since gonadal hormones are widely distributed in the brain and modulate behavior across vertebrate species—from teleosts to primates (reviewed in [431,463,468,471,472,473])—they may play a similar role in lampreys [159]. However, despite the apparent association between gonadal hormones and reproductive functions, their specific identities, receptors, and roles remain poorly characterized in sea lampreys. Further research in both males and females is essential to better understand the endocrine regulation of reproductive behavior in this species.

### 4.3. Section Summary

The neural mechanisms that allow lampreys to adapt their olfactory behavior to environmental factors and biological needs have not yet been revealed. In this section, we propose several mechanisms that could underlie behavioral plasticity in response to odor cues. Potential candidates include variability in olfactory receptor expression within the peripheral olfactory organ and the modulatory effects of neurotransmitters such as GABA, dopamine, and serotonin in the OB. These mechanisms may directly influence OB output, therefore altering motor responses to odors. Additionally, input from other sensory systems shapes olfactory behavior, ensuring adaptation to environmental conditions. For instance, despite detecting migratory pheromones, lampreys suspend migratory activity when exposed to daylight. Moreover, internal signals, including hormones such as lGnRH and gonadal steroids, may further refine olfactory behavior to align with biological needs that vary based on sex and developmental stage. Given the existence of sex- and maturity-specific differences in levels of hormones and their receptors along the olfactomotor pathways, this neural substrate likely contributes to behavioral plasticity in response to odor cues.

The central nervous system serves as the intermediary between sensory inputs and motor outputs, providing a hardwired neural pathway linking odor detection to motor responses [272,294,337]. However, olfactory behavior is not rigid. It varies with environmental conditions, developmental stage, and sex, which implies the presence of modulatory mechanisms along this circuitry. Further studies will deepen our understanding of how these neural mechanisms enable behavioral plasticity throughout the lamprey’s life cycle.

## 5. Conclusions

Lampreys, as one of the most ancient extant vertebrates, provide a unique and evolutionarily informative window into the integration of olfactory inputs with complex behavioral, endocrine, and neuronal responses. Indeed, the study of their well-characterized olfactory system has enabled the identification of specific molecules that induce ecologically relevant behaviors in natural settings. Lampreys rely on olfaction at every significant stage of their life cycle, from larval development through metamorphosis, migration, and reproduction. This review highlights how chemosensory signals elicit robust and context-dependent behavioral reactions such as feeding, predator avoidance, and reproduction. It underscores the ecological importance of olfaction in their life cycle. Moreover, lamprey pheromones initiate profound neuroendocrine changes by activating the HPG axis to mediate physiological transformations critical for reproduction. Here, we proposed a hypothetical endocrine signaling pathway that promotes reproductive fitness and synchrony by accelerating gametogenesis and increasing pheromone production. Finally, we also described the underlying neuronal pathways that convert olfactory inputs into motor output. Lampreys possess a relatively simple yet highly specialized olfactory system that enables the precise detection and processing of chemical signals by specific brain regions, which elicit odor-evoked locomotion. Furthermore, we considered how environmental cues and internal states may exert modulatory influences on these circuits to execute context-appropriate motor behaviors, which highlights the potential for dynamic adjustments in olfactory-driven behavior. Together, these insights illuminate the integrative role of olfaction in lamprey biology and reveal a highly integrated sensory system finely tuned to ecological demands. Continued investigation into this system will be essential for fully unraveling how odors interface with endocrine and neural systems to shape adaptive behaviors.

## Data Availability

No new data were created or analyzed in this study. Data sharing is not applicable.

## Abbreviations

The following abbreviations are used in this manuscript:

AOO	Accessory olfactory organ
DkPES	3,12-diketo-4,6-petromyzonene-24-sulfate
FSH	Follicle-stimulating hormone
GABA	γ-aminobutyric acid
GnIH	Gonadotropin-inhibitory hormones
GnRH	Gonadotropin-releasing hormone
HPG	Hypothalamic-pituitary-gonadal
lGnRH	Lamprey gonadotropin-releasing hormone
LH	Luteinizing hormone
LPal	Lateral pallium
medOB	Medial olfactory bulb
MLR	Mesencephalic locomotor region
MOB	Main olfactory bulb
MOE	Main olfactory epithelium
OB	Olfactory bulb
OR	Odorant receptor
PT	Posterior tuberculum
PZS	Petromyzonol sulfate
RS	Reticulospinal
TAAR	Trace amine-associated receptor-like
V1R	Vomeronasal type 1 receptor
V2R	Vomeronasal type 2 receptor
15α-P	15α-hydroxyprogesterone
3kPZS	3-keto petromyzonol sulfate
3sPZS	Petromyzonol tetrasulfate
